# Fungal exposure in homes of patients with sarcoidosis - an environmental exposure study

**DOI:** 10.1186/1476-069X-10-8

**Published:** 2011-01-20

**Authors:** Marjeta Terčelj, Barbara Salobir, Matevz Harlander, Ragnar Rylander

**Affiliations:** 1 Clinic of Respiratory Diseases and Allergy, University Medical Centre, Ljubljana, Slovenia; 2 BioFact Environmental Health Research Centre, Lerum, Sweden

## Abstract

**Background:**

There is increasing evidence that exposure to moulds (fungi) may influence the development of sarcoidosis. To assess the influence of the environmental exposure, a study was undertaken to determine the exposure to fungi in homes of subjects with sarcoidosis.

**Methods:**

Subjects were patients with clinically established sarcoidosis recruited during the period September 2007 till June 2010. Of these 55 were newly diagnosed and currently under treatment for less than one year, 25 had been treated and had no recurrence and 27 had been treated but had recurrence of the disease. Controls were healthy subjects without any respiratory symptoms (n = 30). Samples of air (about 2.5 m^3^) were taken in the bedroom of the subjects using a portable pump and cellulose ester filters. The filters were analysed for the content of the enzyme N-acetylhexosaminidase (NAHA) as a marker of fungal cell biomass, using a specific substrate and a fluorescent technique and expressed as NAHA units (U)/m^3^.

**Results:**

Compared to controls, subjects undergoing treatment of the disease (newly diagnosed or with recurrence) had significantly higher activities of NAHA in their homes than controls (33.6 and 39.9 vs 10.0 U/m^3^, p < 0.001 and <0.001). Among controls only 5 out of 30 subjects had levels of NAHA above the second quartile value (14 U/m^3^). In homes of subjects with newly diagnosed disease with treatment less than one year, values above 14 NAHA U/m^3 ^were found among 35 out of 55 and among those with recurrent disease among 18 out of 27.

**Conclusions:**

The higher activities of NAHA enzyme found in homes of subjects with active and recurrent sarcoidosis suggest that exposure to fungi is related to the risk of sarcoidosis. Further environmental studies to assess the importance of this exposure for subjects with sarcoidosis are warranted. The results suggest that remedial actions in homes with high levels of fungi may be justified.

## Background

It has been suggested that environmental agents, particularly microbes, influence the risk to develop sarcoidosis [1], Epidemiological studies have shown relationships between sarcoidosis and environments where mould (fungi) would be present. Occupational risk factors were evaluated among 273 cases of sarcoidosis and 618 siblings without the disease [2]. Specific risk exposures that imply possible fungal exposures were vegetable dusts, and indoor exposure to high humidity, water damage or musty odours. In a case-control study on 706 newly recruited cases of sarcoidosis and equal numbers of age-, race-, and gender matched controls, there were positive associations between sarcoidosis and agricultural employment, and work environments with mould/mildew exposure [3]. It has also been reported that sarcoidosis developed among subjects in a group of persons working in a building with severe mould problems [4]. An increase risk for sarcoidosis has also been found among fire-fighters [5,6]. They work in an environment with high levels of aerosolized organic, humidity laden materials.

These findings indicate that fungi in the environment could play a role in the development of the disease. This possible relationship does not necessarily imply infection but could also be the results of an immune reaction to one or several agents in the fungal cell wall. A possible mechanism could be the development of a late hypersensitivity reaction towards some agent in the fungal cell wall such as β-glucan or chitin [7]. β-glucan itself has an immunosuppressive potential [8] and this could induce a pathway of sensitisation to other agents in the fungal cell wall or agents present elsewhere.

If fungi were to play a role for the development of sarcoidosis, one would expect that there are gradients in the environmental exposure between those with the disease and healthy controls. To test this hypothesis, a study was undertaken with the aim to determine airborne levels of fungi in homes of subjects with sarcoidosis and those without pulmonary disease. The presence of fungal cells was determined by analysing the air samples for the enzyme N-acetylhexosaminidase (NAHA) as a marker of fungal cell biomass [9].

## Methods

### Subjects

The clinic of respiratory diseases and allergy at the University medical centre, Ljubljana, Slovenia is one of the national centres for patients with sarcoidosis. For the diagnosis the ERS/ATS criteria [10] are used, comprising a bronchial lung lavage (BAL) and one or more transbronchial biopsies. The routine at the clinic is to make once bronchoscopy with 5 to 10 transbronchial biopsies and BAL with 200 mL. The presence of non-caseating granulomas is verified histologically. If a biopsy is not considered representative, the patient undergoes surgical pulmonary or lymph node biopsy.

Aspiration is performed from one lobe for microbiological analysis of bacteria, fungi and tuberculosis. The BAL fluid is cultured and most biopsies are stained (silver staining, Gomori) to exclude the presence of fungal infection. IgA, IgG and IgG antibodies against Candida and Aspergillus are determined.

Indications for treatment at the clinic are stage II and III with worsening of pulmonary symptoms, including cough and/or shortness of breath or chest pain, and one or more of the following criteria: deteriorating lung function with a fall in total lung capacity of 10 percent or more, a fall in forced vital capacity in one second (FEV_1_) of 15 percent or more, a decrease in diffusing capacity of 20 percent or more, decreased gas exchange at rest or with exercise, progressive radiologic changes or development of signs of pulmonary hypertension. All patients with extra-pulmonary sarcoidosis are treated. Treatment is not given to patients with stage I, II or III who are asymptomatic.

The protocol for treatment at the clinic is methylprednisolone 0.5 mg/kg ideal body weight every day after breakfast during the first for four weeks and then 0.25 mg/kg alternative day therapy during one year.

Subjects for the study were recruited from the sarcoidosis patients during the period September 2005 till June 2010. During this period there were 207 patients (24 stage I, none treated, 142 stage II, 66 treated, 34 stage III, 28 treated, and 6 stage IV, all treated). The subjects were chosen on an *ad hoc *basis when they came to follow-up at the outpatients' care.

We recruited 8 patients with stage I, 71 patients with stage II, 27 patients with stage III and one with stage IV. Extra pulmonary manifestations were present in 27. They were divided into three groups. One comprised subjects with newly diagnosed sarcoidosis and currently undergoing treatment based on the clinical criteria described above. The second group was subjects diagnosed with mild sarcoidosis of all stages, not receiving treatment or who had been successfully treated more than two years earlier and had no evidence of active disease. The third group comprised subjects who failed to respond to treatment for more than one year or had a relapse of the disease after termination of treatment (stage II and III except for one subjects with stage IV). There was only five smokers in the group.

Controls were members of the staff and other contacts who were healthy, non-smoking and without respiratory symptoms and without family members with respiratory disease. The study was approved by the Ethical Committee at the University Medical Centre, Ljubljana (198/05/04) and informed consent was obtained.

Determinations were made of pulmonary diffusion capacity (DL_CO_). serum angiotensin converting enzyme (sACE), chitotriosidase (CTO), and total IgE, using standard laboratory methods. These measurements were not performed among the controls.

Background data for the different groups are shown in Table 1.

**Table 1:** Characteristics of study subjects.

Group	Control	Sarcoidosis		
Parameter		<1 year	no recurrence	recurrence

n	30	55	25	27

Mean age years	46,4 (2.7)	43.2 (1.6)	49.4 (2.6)	47.7 (2.0)

Females %	50.0	55.6	61.9	44.8

DL_CO_%	-	89 (2)	87 (4)	86 (4)

BAL CD4^+^/CD8^+^	-	7.6 (0.9)	5.6 (0.9)	4.6 (0.8)

sACE μKat/L	-	0.43 (0.03)	0.44 (0.04)	0.38 (0.05)

CTO nmol/h/mL	-	632 (86)	523 (91)	513 (97)

Total IgE mg/mL	-	98 (26)	125 (50)	313 (197)

Extrapulmonary manifestations	-	25	8	12

Mean and SEM.

The groups were relatively comparable although the mean age was slightly lower in the group under treatment and the proportion of females was slightly higher in the group with no recurrence. The CTO value was slightly higher in the group under treatment than in the other groups (NS). The IgE value was higher in the group with recurrence as compared to the other groups (NS). Extra pulmonary manifestations were slightly more frequent in the group < 1 year than in the other groups (NS).

### Air sampling

The participants were supplied with a portable pump equipped with filter holders preloaded with cellulose acetate filters (PCM Cassettes with mixed Cellulose Esters Filter, 25 mm, 0.8 μm pore size, Zefon International, Inc., Ocala, FL, USA). The subjects turned on the pump and sampling was performed during about four hours. The windows and doors to the room were closed several hours prior to the sampling. The exact volume sampled was read from a volume meter attached to the pump and was usually about 2.5 m^3^.

### Enzyme analysis

For the enzyme analysis one mL of a fluorogenic enzyme substrate (4-methylumbelliferyl N-acetyl-beta-D-glucosaminide, Mycometer A/S, Copenhagen, Denmark) was added to the filter and incubated for around 30 minutes - the exact time being set by the room temperature. Thereafter 2 mL of an alkaline buffer (the developer) was added to the filter. The liquid in the filter holder was sucked out through the filter and collected in a cuvette. The fluorescence of this fluid was read in a fluorometer (Picofluor, Turner Designs, Sunnyvale, CA, USA) and the fluorescence value for a control filter was deducted from the sample value. One count is equal to 2.3 ng Apergillus oryzae NAHA. To avoid random scatter, the values read in the fluorometer were divided by 10 and given as a round figure to express the NAHA enzyme activity in units (U/m^3^).

The reproducibility of the sampling is very high. With two filters samples taken in parallel in 25 different rooms, the R^2 ^was 0.956 with ANOVA f 504.2, p < 0.001. For samples taken on two occasions a few weeks apart in 26 rooms, the R^2 ^was 0.796 with ANOVA f 93.42, p < 0.001. Repeated readings of the same sample on the fluorometer showed very small variations in the order of a few units.

### Statistical analysis

Differences between groups were evaluated for statistical significance using the Mann-Whitney and the chi^2 ^tests. Relationships were calculated using Spearman's test. A p-value of 0.05 was considered significant.

## Results

The activities of NAHA in the different groups are shown in Table 2.

**Table 2:** NAHA U/m^3 ^in rooms of subjects in different groups of sarcoidosis. Mean and SEM.

Group	control	< one year	no recurrence	recurrence
n	30	55	25	27
NAHA	10.0 (1.1)	33.6 (5.0)	12.0 (1.8)	39.9 (7.6)

Subjects under treatment or with recurrence had significantly higher values than controls and those who were treated and had no recurrence (p < 0.001, Mann-Whitney).

The NAHA values were highly skewed and the values from the whole material (controls and sarcoidosis) were thus divided into those below or above the second quartile (14 NAHA U/m^3^). Table 3 shows the number of controls and cases of sarcoidosis in the low and high exposure groups.

**Table 3:** Presence of low and high activities of NAHA (below or above the 2^nd ^quartile, 14 NAHA U/m^3^) in homes of controls and patients without and with recurrence of sarcoidosis.

*Group*	*n*	*low*	*high*
Controls	30	25	5
Sarcoidosis			
- treatment < 1 year	55	20	35
- no recurrence	25	16	9
- recurrence	27	9	18

The difference in distribution between controls and subjects under treatment less than a year was significant (p < 0.001) as well as that of controls vs recurrence (p < 0.001, chi^2 ^test). For those with no recurrence, there was no difference to controls.

## Discussion

The major result from the study is that high activities of an airborne enzyme indicating fungal cell mass were found significantly more often in the homes of subjects with ongoing or recurrent sarcoidosis.

There are some limitations in the study. The measurements were made on one occasion only. For a complete environmental assessment, measurements should also have been made at the workplace or in other environments where the subject spent considerable time. On the other hand experience from a previous study [11] suggests that mould levels in a certain room vary relatively little within a year, particularly in buildings with high levels of moulds. Provided that windows and doors in the room where measurements were made are closed prior to the sampling, the values reflect the exposure of the inhabitants, irrespectively of outdoor conditions.

In spite of these shortcomings considerable differences were found between the level of airborne enzyme in homes of persons with sarcoidosis and control subjects. There was a larger proportion of subjects with high activities of NAHA in their homes among those with recurrent sarcoidosis. From an exposure point of view this is logical, as a continuous exposure to a causative agent would represent a risk for recurrence, as compared to an exposure that occurred during a limited time period and was no longer present when the disease had been treated. The reason why subjects with a history of disease but no recurrence had low values could be due to cleaning of the home, remediation of building related humidity problems or moving to another location, No systematic evaluation of these possibilities was made in this study,

The analysis of airborne samples comprised the determination of NAHA as a measure of fungal biomass. Measurements of fungal cell agents are more precise than ocular inspection to detect fungi [12]. Previous studies have reported that NAHA represents fungal biomass both in the growth and stationary phase [13-15]. Significant correlations between NAHA and total spore counts were found in air samples and in dust generated from biomass in a biofuel plant [16,17]. Strong correlations were found between fungal biomass (gravimetric weight) and NAHA in fungal species grown on nutrient agar and between ergosterol and NAHA activity on mould contaminated gypsum boards [9]. A linear correlation between NAHA and Aspergillus niger biomass has been reported [18].

NAHA is produced by a range of organisms including bacteria, fungi, protozoa, and mammalian cells and is thus not specific for fungi. On the other hand when the fungi grow indoors such in humid conditions, there is no reason to believe that other sources of NAHA such as pollen or mammalian cells would also multiply. A previous study has demonstrated that the sensitivity and specificity for detection of buildings with fungi are very high [19] and the possible confounding sources of NAHA did not play an important role. Although bacteria are generally present in environments with fungal growth, their cell wall agents such as endotoxin elicit other kinds of immunological responses than the granulomatous inflammation induced by fungal components such as β-glucan.

None of the cases of sarcoidosis investigated had any signs of fungal infection as evidenced by low titres against common fungi and negative cultures of lung biopsies or lung lavage. This suggests that, if fungi contribute to the development of sarcoidosis, the effect would be mediated by other mechanisms than infection. There are several fungal cell components which may influence the innate immune system. For one of these - β-glucan - information is available both from experimental and field studies [8]. At low doses, similar to those present in the environment, the major initial effect is an immune suppression and a Th2 deviated lymphocyte response which may deviate to a Th1 driven response with repeated exposures [20]. This effect would be present even if the fungal cell is dead as β-glucan is known to remain in tissue several weeks after the disintegration of the fungal cell itself [8].

The proportion of patients with high activities of NAHA in their homes was larger in the group with recurrent disease. This suggests that they were continuously exposed to fungi. Cleaning of fungal growth at home might thus be a measure to prevent the recurrence of sarcoidosis.

The indoor environment comprises exposure to a variety of agents some of which have the potential to cause irritation in the airways. Even if the primary agent studied here was fungal cell mass, it cannot be excluded that agents such as cooking fuels or environmental tobacco smoke might influence the exposure outcome and hence the risk of disease. Nutritional factors are an example of another important environmental influence. Antioxidant status has also been associated with sarcoidosis although it is unclear if it is a consequence of the disease [21].

## Conclusion

Measurements of the enzyme NAHA as a marker for fungal biomass demonstrated significantly higher activities in homes of subjects with sarcoidosis, particularly among those with recurrent disease. This suggests that exposure to fungi contributes to the pathology of the disease. Evaluations of fungal exposure in the environment of patients with sarcoidosis should be considered, particularly for those with recurrent sarcoidosis. Routine examination of sarcoidosis patients should include information on potential fungal exposure at home or at the workplace and cleaning actions could be suggested.

## Abbreviations

NAHA: N-acetylhexosaminidase; CTO: chitotriosidase; sACE; serum angiotensin converting enzyme; BAL: brochoalveolar lavage; FEV_1_; forced expiratory volume in one second; NS: non significant

## Competing interests

The authors declare that they have no competing interests.

## Authors' contributions

M.T., B.S., and M.H. were responsible for the clinical issues. R.R. was responsible for the NAHA measurements and the manuscript structure. All authors contributed to the data analysis and have read and approved the final manuscript.
